# The role of platelets in the recruitment of leukocytes during vascular disease

**DOI:** 10.3109/09537104.2015.1064881

**Published:** 2015-07-21

**Authors:** G. Ed Rainger, Myriam Chimen, Matthew J. Harrison, Clara M. Yates, Paul Harrison, Stephen P. Watson, Marie Lordkipanidzé, Gerard B. Nash

**Affiliations:** ^a^ Centre for Cardiovascular Sciences, Institute for Biomedical Research, The Medical School, The University of Birmingham, Birmingham, UK; ^b^ Universite de Montreal, Faculte de Pharmacie, Montreal, Quebec, Canada

**Keywords:** Adhesion, inflammation, leukocytes, platelets, vascular disease

## Abstract

Besides their role in the formation of thrombus during haemostasis, it is becoming clear that platelets contribute to a number of other processes within the vasculature. Indeed, the integrated function of the thrombotic and inflammatory systems, which results in platelet-mediated recruitment of leukocytes, is now considered to be of great importance in the propagation, progression and pathogenesis of atherosclerotic disease of the arteries. There are three scenarios by which platelets can interact with leukocytes: (1) during haemostasis, when platelets adhere to and are activated on sub-endothelial matrix proteins exposed by vascular damage and then recruit leukocytes to a growing thrombus. (2) Platelets adhere to and are activated on stimulated endothelial cells and then bridge blood borne leukocytes to the vessel wall and. (3) Adhesion between platelets and leukocytes occurs in the blood leading to formation of heterotypic aggregates prior to contact with endothelial cells. In the following review we will not discuss leukocyte recruitment during haemostasis, as this represents a physiological response to tissue trauma that can progress, at least in its early stages, in the absence of inflammation. Rather we will deal with scenarios 2 and 3, as these pathways of platelet–leukocyte interactions are important during inflammation and in chronic inflammatory diseases such as atherosclerosis. Indeed, these interactions mean that leukocytes possess means of adhesion to the vessel wall under conditions that may not normally be permissive of leukocyte–endothelial cell adhesion, meaning that the disease process may be able to bypass the regulatory pathways which would ordinarily moderate the inflammatory response.

## Introduction

It is now widely recognised that platelets play an import role in the pathology associated with cardiovascular diseases. The literature on the contribution of platelets to symptomatic atherothrombotic disease, which is predicated on the formation of a mural thrombus in diseased arteries, is extensive. As this aspect of platelet biology is well developed and is already the target of a number of successful and well validated therapeutic pathways, we do not propose to address it in the current discussion. The reader is directed to a recent review of this area for additional information [1].

Here, having briefly revisited endothelial cell (EC) mediated pathways of leukocyte recruitment during inflammation, the roles of platelets in the recruitment and function of leukocytes in cardiovascular diseases will be discussed. In particular, the molecular basis of the interactions between platelets, leukocytes and the vessel wall are reviewed. The ability of immobilised platelets to recruit leukocytes is well established and discussed in detail. However, the developing field of leukocyte–platelet aggregation in circulating blood is also addressed, including emerging paradigms on the importance of platelet microvesicles (PMV) in the regulation of leukocyte recruitment in cardiovascular diseases. Finally, the current therapeutic options for intervening in the platelet-mediated recruitment of leukocytes in cardiovascular diseases will be discussed.

## Leukocyte adhesion during resolving inflammation

In vertebrates, infection or tissue trauma cause the rapid initiation of an acute and resolving inflammatory response. The identity of the leukocytes within the tissue infiltrate vary with time and throughout the evolution of the inflammatory response in order that the requirements for repair of the affected tissues and the development of an adaptive immune response are matched to the specialised functions of the different leukocyte subsets [2].

**Figure 1.**
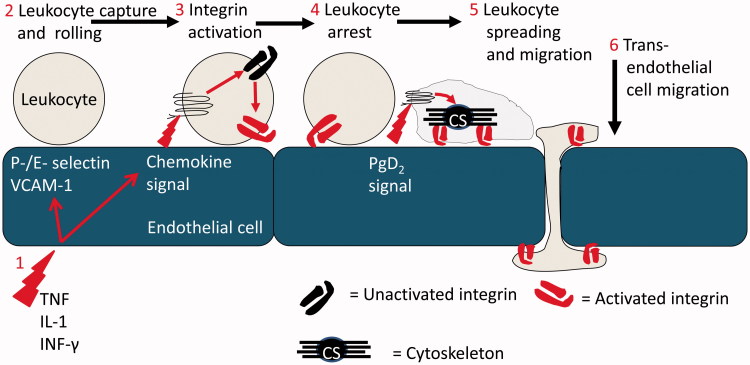
The multi-step leukocyte adhesion cascade: (1) Endothelial cells at a site of inflammation are activated by stromal derived inflammatory cytokines such as TNF-α, IL-1β and IFN-γ. Induction of transcriptional activity results in the expression of adhesion molecules and chemokines which coordinate leukocyte recruitment. (2) Leukocytes are recruited from flowing blood by specialised receptors of the selectin family and VCAM-1, which also support rolling adhesion. (3) Chemokine signals activate the β1 and β2 integrins on rolling leukocytes. (4) Adhesion is stabilised and the leukocytes become firmly adherent to the endothelial cell. (5) In response to additional signals, e.g. from PgD2, the leukocyte cytoskeleton undergoes remodelling driving shape change (spreading) and dynamic integrin-mediated migration. (6) Leukocytes migrate across and through the endothelial cell monolayer and onwards into tissue.

The molecular processes that support leukocyte recruitment in post-capillary venules (the site where leukocytes are recruited during acute inflammation) are well described (Figure 1). Thus, local inflammatory mediators, such as histamine or cytokines (e.g. tumour necrosis factor-α – TNF), activate ECs resulting in the expression of specialised adhesion receptors that form short lived bonds with counter ligands on the leukocyte surface [3–5]. Thus, E- and P-selectin and the immunoglobulin super-family (IgSF) member, vascular adhesion molecule-1 (VCAM-1), are the major receptors that tether leukocytes from rapidly flowing blood [6]. The sequential formation and dissolution of these bonds supports a characteristic and dynamic form of adhesion, referred to as rolling [7] which does not require leukocyte activation. However, leukocyte migration through the vessel wall and into the inflamed tissue is dependent upon the receipt of an activating stimulus [7, 8]. Peptide agonists of the chemokine family are the major activating stimuli presented to rolling leukocytes in order to stabilise adhesion [9, 10], although numerous other mediators have been reported to play a role in leukocyte recruitment under some inflammatory conditions (e.g. platelet activating factor [PAF] and leukotriene-B4 [LTB4]) [11–14]. These signals result in remodelling of the cytoskeleton and activation of the β1 and β2-integrin adhesion molecules that provide firm adhesion. The integrins also support apical and trans-EC migration of adherent leukocytes using intercellular adhesion molecule-1 (ICAM-1) and VCAM-1 as counter ligands [9]. Efficient onward migration after chemokine activation also requires additional downstream signals. Thus, neutrophils and T-lymphocytes require a prostaglandin-D2 (PGD2) signal to undertake diapedesis *in vitro* [15]. In addition, molecules at the EC junction have been implicated in the regulated trafficking of leukocytes, including CD31 (platelet-endothelial cell adhesion molecule-1 – PECAM-1), CD99 and family members of the junctional adhesion molecules (JAM) family, e.g. JAM-C [16, 17].

## Platelets are a sufficient substrate to support the recruitment, activation and migration of blood borne leukocytes

There is now an increasing awareness that aberrant pathways of leukocyte recruitment might be established in disease states [18]. Of relevance here was the increasing realisation that platelets might promote leukocyte adhesion during inflammation, in particular the concept that platelet interactions with the vessel wall might lead to inappropriate leukocyte recruitment in atherosclerosis. Such hypotheses were predicated on the assumption that activated platelets were capable of recruiting leukocytes from the blood. This was a reasonable expectation, as platelet α-granules contain P-selectin which is expressed upon activation [19, 20]. In addition, they release leukocyte activating agents (see below for details).

Early studies showed conclusively that monolayers of immobilised, spontaneously activated platelets were able to recruit flowing leukocytes using P-selectin [21, 22]. Both granulocytic and mononuclear leukocytes were captured efficiently from flow and could roll continuously across the monolayer without apparent signs of activation. Importantly, if the platelets were activated by thrombin (i.e. through protease-activated receptors [PAR] – PAR1 and PAR4), neutrophils were rapidly activated through the CXCR2 receptor, putatively by platelet-derived CXCL7 (NAP-2) [23]. Other studies using activated platelets have identified alternative pathways of neutrophil activation. Thus, thrombin stimulated platelets activate neutrophils through PAF and LTB4 [24], while platelets bound to and activated by collagen type 1, activated neutrophils through PAF [25]. Interestingly, LTB4 is not a platelet-derived eicosanoid, but can be synthesised by neutrophils from platelet-derived arachidonic acid [26], making it an exemplar of transmetabolic activity requiring the integrated activity of the haemostatic and immune systems. Other CXC chemokines have been reported in platelets, although their function in leukocyte recruitment remains undetermined. Thus, a full-length cDNA for CXCL5 (ENA 78) was cloned from human platelets [27], although transcribed protein was not measured. In addition, rabbit platelets contain preformed and stored CXCL8 (IL-8), which was secreted upon platelet activation [28].

Using activated neutrophils, the β2-integrin, CD11b/CD18 (αMβ2; Mac-1) supported immobilisation on the platelet monolayer using platelet fibrinogen as a counter ligand [29, 30]. Interestingly, immobilised platelets could also provide signals through specific receptors (e.g. P-selectin and CD31) that regulated the velocity and direction of neutrophil migration [31, 32]. Moreover, neutrophils migrating on a platelet monolayer formed large embolising aggregates when they came into contact with each other [33]. Diacovo et al. showed that platelets immobilised on fibronectin-coated polycarbonate filters, supported neutrophil migration towards an exogenous chemotactic agent added below the filter [29].

## Platelets can bind to appropriately stimulated endothelial cell monolayers

**Figure 2.**
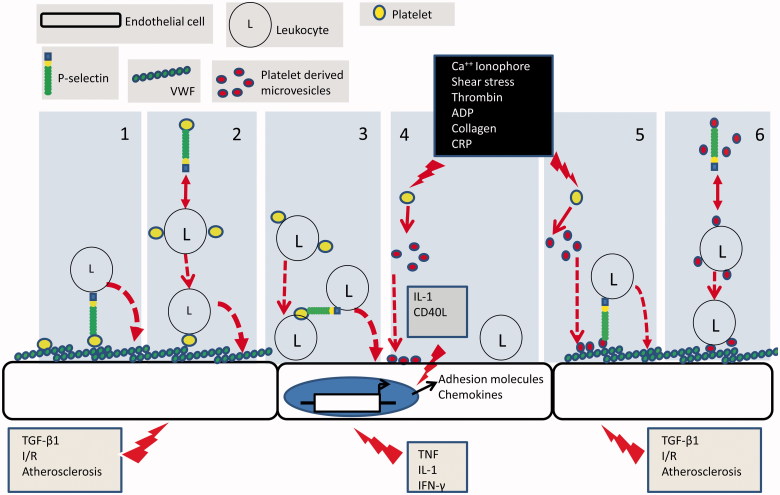
Mechanisms of platelet-mediated leukocyte recruitment: (1) appropriately activated endothelial cells express a matrix of VWF on their surface to which platelets are recruited and activated. Platelet presented P-selectin then forms an adhesive bridge between the endothelial surface and blood borne leukocytes. (2) Platelets form heterotypic aggregates with leukocytes in the circulation in a P-selectin dependent manner. Aggregates are then recruited to the VWF on the surface of appropriately activated endothelial cells. (3) Inflammatory cytokines induce transcriptional programmes in endothelial cells which result in the expression of adhesion molecules and chemokines that support leukocyte adhesion. Leukocytes in heterotypic aggregates which are recruited to the endothelial cell surface posses additional platelet borne receptors. These can in turn support the secondary adhesion of un-aggregated leukocytes. (4) Upon activation platelets shed microvesicles which bind directly to endothelial cells. Microvesicle borne inflammatory cytokines induce transcriptional programmes in endothelial cells which result in the expression of adhesion molecules and chemokines that support leukocyte adhesion. (5) P-selectin bearing PMV bind to appropriately activated endothelial cells. Platelet presented P-selectin then forms an adhesive bridge between the endothelial surface and blood borne leukocytes. (6) P-selectin bearing PMV form heterotypic aggregates with leukocytes in the circulation. Aggregates are then recruited to VWF on appropriately stimulated endothelial cells utilising microvesicle borne adhesion receptors.

The concept that platelets may interact directly with ECs conditioned by an inflammatory environment has received increasing attention. Ordinarily, ECs present an anti-thrombotic surface to flowing blood by releasing nitric oxide (NO) and prostacyclin (PGI2) and through expression of CD39 [34]. Signalling into the platelet by PGI2 and NO maintains high levels of cyclic nucleotides within the platelet cytoplasm, thereby antagonising all the known pathways of platelet activation [35–37], while the ectonucleotidase activity of CD39 enzymatically degrades ADP, one of the two major feedback agonists involved in platelet activation. Early work thus assumed that EC ‘damage’ was required to expose matrix to which platelets could bind [38, 39]. This paradigm has been modelled *in vitro* using sub-confluent ECs, where matrix proteins readily recruit platelets from perfused blood [40]. However, more recent *in vitro* studies show that platelets can adhere directly to endothelium when either the ECs and/or platelets are activated by agents such as thrombin or ADP (platelets) or the cytokines IL-1β, TNF-α or TGFβ1 (EC) [39, 41–54]. Figure 2 shows the routes by which platelets may facilitate the recruitment of leukocytes to the vessel wall that are discussed in this review.

Intravital experiments in mice showed platelet adhesion to mesenteric venules following treatment with calcium ionophore and to arteries at sites of atheroma formation [55–57]. In addition, stimulation of the murine cremaster muscle for 4 hours by topical application of TGF-β1 results in platelet deposition in post-capillary venules [45]. Platelet adhesion in these models was supported by bridging of platelet αIIb/β3 integrin to EC αvβ3 integrin by von Willebrand factor (VWF) [41, 42, 44, 49, 52, 55] with a possible contribution from P-selectin [58]. An interesting mechanism for delivering platelets to EC was reported by Van Gils et al. [59] who showed that platelets in aggregates with monocytes (a phenomenon discussed in detail below) relocated to the rear of the leukocyte before detaching and adhering to an EC upon monocyte diapedesis. One can speculate that this process might be relevant to the secondary recruitment of blood borne leukocytes to the vessel wall during inflammation.

## Platelets adherent to the vessel wall can recruit blood borne leukocytes

The concept that platelet deposition to the vessel wall can promote the recruitment of leukocytes now has substantial experimental support. *In vitro*, numerous studies demonstrate platelet-mediated leukocyte recruitment to adhesive substrates of matrix proteins or ECs. Thus, when whole blood was perfused across sub-confluent cultures of ECs, platelets bound to exposed matrix and subsequently supported the recruitment and activation of perfused neutrophils [60]. In flow based models of vascular damage, platelets deposited from whole blood onto collagen could recruit leukocytes [61, 62], and this was most efficient under conditions of disturbed flow in a model recapitulating the environment found downstream of atherosclerotic plaque. In this model, platelets and leukocytes were delivered to the substrate in a continuously rotating vortex which developed downstream of a step in the flow chamber [63]. Using whole blood under flow conditions, even platelets that were sparsely adherent to ECs could efficiently recruit neutrophils and monocytes [45, 61]. Interestingly, secondary recruitment of monocytes to ECs was supported by platelet-monocyte aggregates that were adherent to the endothelium, with platelet P-selectin acting as a bridge to deliver flowing monocytes to the EC [64]. Where it was characterised in these studies, authors showed that leukocyte capture was mediated by platelet P-selectin and stable adhesion was supported by leukocyte β2-integrins.

An important aspect of these studies is the diversity of leukocytes recruited by vessel wall adherent platelets. Thus, these as well as other reports [65, 66] detailed the platelet-mediated recruitment of neutrophils, monocytes, dendritic cells, T-lymphocytes, B-lymphocytes and NK-cells to endothelium. This lack of specificity is not surprising, as the counter ligand for P-selectin, P-selectin glycoprotein ligand-1 (PSGL-1), is present on many leukocyte subsets. However, a number of observations describing specificity in the platelet-mediated recruitment of leukocytes are of particular interest. Kuckleberg et al. [45] demonstrated that monocytes were preferentially recruited by EC adherent platelets in an EC-smooth muscle cell co-culture model of the atherosclerotic artery wall. The ECs were activated by TGF-β1 generated in co-culture, and could recruit flowing platelets to surface VWF, a model that could be recapitulated by activating ECs with recombinant TGF-β1. Specificity for monocytes arose through EC production of CCL2 (MCP-1), which preferentially stabilised the adhesion of monocytes captured from flowing whole blood by platelet P-selectin. Two other groups using intra-vital observations in atherosclerotic mice (*ApoE−/−*) also observed chemokine dependent specificity of monocyte adhesion to the carotid artery [56, 67]. In these studies, adhesion of platelets which presented P-selectin, and deposition of platelet-derived CCL5 (Regulated on Activation, Normal T cell Expressed and Secreted – RANTES) and CXCL4 (platelet factor-4 – PF-4) onto the endothelium, was responsible for increased monocyte adhesion. Schulz et al. demonstrated a similar paradigm on cytokine stimulated human ECs [68]. Here, platelets binding from whole blood captured leukocytes through P-selectin and monocytes were subsequently activated by EC derived CX3CL-1 (fractalkine). In addition, blockade of CX3CR1 by injection of a function neutralising antibody into the *ApoE−/−* mouse, inhibited the adhesion of WEHI-274.1 cells (a monocytic cell line) to the carotid artery. Thus, although the initial tethering of leukocytes by platelet P-selectin may not be selective, specificity for recruitment of leukocyte subsets, in particular monocytes can be achieved by the presentation of chemokines, the receptors for which are restricted to a limited repertoire of leukocytes.

## The formation of heterotypic leukocyte–platelet aggregates in the blood

The formation of platelet–leukocyte aggregates in the blood, and the subsequent presentation of platelet adhesion receptors on the leukocyte, would mean that circulating leukocytes would possess a means of adhesion to the vessel wall that is not normally present during inflammation. The formation of heterotypic platelet–leukocyte aggregates in blood is dependent upon platelet activation [69]. This leads to platelet P-selectin binding to leukocyte PSGL-1 to establish heterotypic adhesion [70]. Indeed, blocking the function of P-selectin or PSGL-1 with neutralising antibodies is an effective means of ablating aggregate formation [70–72]. Importantly, intercellular adhesion is further stabilised directly or indirectly (via fibrinogen bridging) by other receptors, e.g. GPIb and αIIbβ3 integrin on platelets and the β2 integrins (in particular, CD11b/CD18) on leukocytes [73, 74].

The formation of platelet–leukocyte aggregates occurs with a low incidence even in healthy individuals. Indeed, platelet–monocyte aggregates appear to be more common in healthy children than in healthy adults [75], indicating that their occurrence might change with age. Interestingly, there is a positive correlation between the number of circulating leukocyte–platelet aggregates and the severity of inflammatory and infectious diseases. Thus, an increase in aggregates is reported in diabetes and rheumatoid arthritis, diseases associated with accelerated formation of atheroma and increased risk of athero-thrombotic disease [76–78]. An increase in aggregates has also been observed in inflammatory bowel disease [79] and ischaemic heart failure [80]. Interestingly, their incidence is also increased in bacterial infections [81]. Indeed, monocytes with adherent platelets are recruited to sites of *Leishmania major* infection [81], while *Staphylococcus aureus* promotes the formation of neutrophil–platelet aggregates in the circulation [82].

The formation of platelet–leukocyte aggregates (in particular platelet-monocyte aggregates) occurs in atherosclerosis and athero-thrombotic disease. Thus, in both stable coronary artery disease or during coronary or cerebral infarction, the number of circulating aggregates increases significantly [83–89]. Moreover, the incidence of aggregates increases with independent risk factors for cardiovascular and cerebrovascular disease, such as hypertension [90, 91]. Importantly, increases in circulating platelet–monocyte aggregates may themselves be an independent risk factor for cardiovascular and cerebrovascular disease, or at least, their abundance can be used to stratify patients on the basis of predicted outcome after acute myocardial infarction [92]. Interestingly, experiments tracking autologous infused biotinylated platelets (activated *ex vivo*) in animal models demonstrate that the number of circulating monocyte–platelet aggregates may be a more sensitive indicator for *in vivo* platelet activation than circulating neutrophil-platelet aggregates or P-Selectin-positive non-aggregated platelets. Indeed, it has been demonstrated that after percutaneous coronary intervention (PCI), there is an increased number of circulating monocyte–platelet aggregates (and, to a lesser extent, neutrophil-platelet aggregates), but not P-selectin-positive platelets in peripheral blood. These data imply a rapid and efficient association between circulating leukocytes and activated platelets, making the later difficult to observe.

Thus, these studies indicate that heterotypic platelet–monocyte aggregates are associated with the progression of atherosclerotic disease and may contribute to the severity of symptomatic athero-thrombotic disease. However, it is notable that a direct role of heterotypic aggregate formation in the recruitment of monocytes (or other leukocytes) to ECs overlying atherosclerotic plaque has yet to be described.

## The role of PMV in promoting leukocyte recruitment

Microvesicles are heterogeneous plasma membrane derived particles, 100–1000 nm in diameter, which can be detected in blood, urine and other bodily fluids [93]. They are released from cells of the vasculature, including platelets, ECs and leukocytes, and specific populations can be identified using appropriate methodology (e.g. flow cytometry), as they express surface markers derived from their cell of origin. There is mounting evidence that microvesicles play a physiological role in inflammation [94].

Microvesicles are released from activated cells and inflammatory diseases are associated with increased numbers of circulating microvesicles [95]. Microvesicles may be incorporated directly into the plasma membrane [96] or internalised by a recipient cell [97], thereby delivering vesicle contents. As microvesicles transport a wide range of molecular cargo, including DNA [98], mRNA [99], micro-RNA [100], membrane and signalling lipids [101] and proteins [102], there is the potential for important modification of recipient cell function. Importantly, this can include alterations in their adhesion to other cell types.

Microvesicles derived from activated platelets (PMV) are considered to be the most abundant population in human blood [103]. *In vitro*, PMV have been generated in response to shear stress, thrombin, calcium ionophore, ADP, collagen and collagen related peptide [104–107]. Interestingly, proteomic studies on populations of microvesicles derived using different platelet agonists demonstrate the formation of heterogeneous populations. Thus, using thrombin or shear stress as distinct routes to platelet activation, resulted in microvesicles that differed in abundance, as well as the identity of their protein cargos [107]. Importantly, megakaryocytes, which are the bone marrow progenitor cells from which platelets mature, also release microvesicles into the blood. This population of microvesicles shares surface markers such as CD41 (αIIb-integrin subunit) and CD42b (GPIb) with PMV [103]. Thus, studies that use these molecules to discriminate a platelet-derived population of microvesicles should be interpreted with caution, as it is possible that the predominant population of microvesicles expressing CD41 and CD42b are derived from megakaryocytes. Only upon activation do platelets release microvesicles bearing CD62P (P-selectin) which may discriminate the origin of these populations.

An increased incidence of circulating PMV is associated with a number of diseases. In diabetic retinopathy, the number of PMV was associated with the severity of disease [108], while the levels of PMV circulating in type-1 diabetics correlated with the degree of pro-atherogenic dyslipidaemia [109]. There was a correlation with vascular dysfunction (assessed by measuring arterial elasticity and flow-dependent vasodilatation of the brachial artery) [110] in patients with Type-2 diabetes. Interestingly, the number of PMV was higher in patients with acute coronary syndromes than those with stable angina [111], implying an association with the onset of athero-thrombotic disease.

The roles of PMV in inflammation and pathogenesis of inflammatory disease is not well understood. However, they possess adhesion receptors such as GPIb, αIIbβ3-integrin and P-selectin, meaning that they could interact with the vessel wall and circulating leukocytes to promote recruitment of the later. Surprisingly, their role in leukocyte adhesion is sparsely reported. Tersteeg et al. [112] showed that platelets adherent to physiological substrates *in vitro* and *in vivo* elaborated extensive (100’s μm) flow-induced membrane protrusions (FLIPRs) which delivered microvesicles to neutrophils and monocytes rolling in a P-selectin dependent manner. These interactions lead to leukocyte activation using increased CD11b expression and the shedding of CD62L (L-selectin) as markers. The interaction of PMV with ECs has also been studied. Mause et al. [113] demonstrated that PMV rolled on IL-1β stimulated human microvascular ECs using P-selectin and GP1b. This was sufficient for the deposition of microvesicle derived CCL5 onto the ECs, leading to increased arrest of perfused monocytes [113]. Barry et al. [114, 115] observed that the adhesion of monocytes and the monocytic leukaemia cell line, THP-1, to ECs treated with PMV was increased, an outcome that was attributed to the activation of both ECs and leukocytes by the delivery of arachidonic acid (AA) from the microvesicles. Lindemann et al. showed that activated platelets could synthesise IL-1β which was incorporated into PMV [116]. Importantly, IL-1β-mediated activation of ECs by these microvesicles resulted in neutrophil adhesion, although the molecular basis of recruitment was undescribed. PMV also up-regulated adhesion receptors (including E-selectin and ICAM-1) on ECs [100]. PMV also interact directly with leukocytes. Consequently, the expression of adhesion receptors such as CD11b and CD32 and secretion of TNF-α, IL-1β and IL-6 were up-regulated in THP-1 cells [106]. Furthermore, P-selectin expressed on PMV acted as an adhesive bridge between HL-60 neutrophilic cells, leading to the formation of homotypic leukocyte aggregates in flow [117].

Thus, it is clear that PMV increase in the circulation during inflammatory disease. However, at present, it is unknown whether this is a cause or consequence of pathology. Nevertheless, PMV appear to have the potential to amplify inflammation by virtue of the interaction with both leukocytes and ECs.

## The role of platelets in leukocyte recruitment in IR injury

Ischemia and reperfusion (IR) injury is a key contributor to the pathology of cardiovascular and cerebrovascular infarction (i.e. stroke and heart attack). It may also occur as a complication of reconstructive vascular surgery or organ transplantation. It is widely recognised that reperfusion of ischemic tissue exacerbates tissue damage by inducing a marked inflammatory response [118]. It is thus pertinent to consider the role of platelets in leukocyte recruitment, a process which is fundamental to the progression of IR injury [119–122].

Using electron microscopy and immuno-histochemistry, Cywes et al. [123, 124] demonstrated the involvement of platelets in IR injury. They noted platelet accumulation in rat and human livers subject to cold perfusion as a means of preservation prior to transplant (otherwise called preservation injury), a process that imposes an ischaemic insult on the tissue which is reperfused upon transplant. Since then, platelets have been implicated in the progression of IR injury during transplant of other organs including the heart [125], lung [126], liver [124, 127] and pancreas [128]. Ischemia and reperfusion leads to the activation and accumulation of platelets within vascular beds early after reperfusion [129], and platelets are among the first cells recruited to the site of injury [130]. Intravital microscopy has demonstrated that platelets interact with numerous vascular beds in the mouse liver (arterioles, sinusoidal capillaries and venules) after ischemia followed by as little as 20 minutes of reperfusion. The injury induced resulted in hepatocellular and microvascular injury and failure of sinusoidal perfusion [127, 131, 132]. Interestingly, the extent of platelet recruitment to the vascular endothelium is dependent upon the duration of ischemia, and not directly to the duration of reperfusion [127]. P-selectin plays an important role in the adhesion of platelets to the vessel wall during post-ischeamic reperfusion [58, 130, 131, 133–136]. However, the source of P-selectin which mediates these processes is unclear. Different studies indicate that EC [58, 130, 132, 134] or platelet-derived P-selectin [133] or both pools of the molecule [133] are involved in the recruitment of platelets to ECs. Khandoga et al. [131] described an interaction between fibrinogen deposited on ICAM-1 of post-ischaemic ECs, which resulted in platelet deposition in the liver [131], while a number of studies identified platelet αIIbβ3 as the counter ligand for fibrinogen deposited in arterioles and venules following ischemia of different tissues [133, 137–139]. Others have shown roles for reactive nitrogen species [140], angiotensin II type 1-receptors [AT(1)] [141], endothelin-A [142] and lymphocyte derived IFN-γ [143] in promoting the adhesion of platelets to the post-ischaemic vasculature.

There is also substantial evidence that platelets are responsible for a proportion of the leukocyte recruitment observed after reperfusion of ischemic tissue. Thus, Lefer et al. [144] showed that platelets were important for neutrophil recruitment during reperfusion of isolated rat hearts that had been subject to ischaemia. A number of studies using intravital imaging of the vasculature after ischaemia and reperfusion showed that platelets were important for leukocyte recruitment in the microcirculation of the mesentery [145], the small intestine [146], in cerebral vessels, as well as in the retina [134]. Not surprisingly, they all reported a central role for P-selectin in the recruitment of leukocytes.

Platelet-mediated leukocyte adhesion has also been directly implicated in the process of reperfusion injury. Thus, platelets contributed to neutrophil-mediated cardiac dysfunction of isolated perfused rat hearts when mixtures of these cells were perfused after ischaemia [144]. Importantly, platelet depletion during IR preserves organ function in a number of models. Thus, the canine pancreas [128] and liver [147] benefited from such a strategy. Singbartl et al. [148] showed that platelet P-selectin was necessary for neutrophil-mediated acute ischemic renal failure in mice. In addition, in rats, the retinal microcirculation was spared if animals were rendered thrombocytopenic 4 hours prior to ischemia and reperfusion [134].

Thus, platelets are efficiently recruited during the reperfusion of ischemic tissue, and play a role in the recruitment of inflammatory leukocytes, which in turn are responsible for tissue damage. Moreover, blockade of platelet adhesion to post-ischemic vessels, their removal from the circulation prior to ischaemia, or the inhibition of platelet borne P-selectin, can significantly attenuate the severity of reperfusion injury. However, other studies have shown protective effects of platelet-neutrophil aggregate in resolution of inflammatory responses in the microcirculation during IR [149]. Indeed, it appears that platelet–neutrophil interactions allow release of the pro-resolving lipid, lipoxin A4 via fpr2/3, which controls formation of platelet–neutrophil aggregates. Similarly, production of maresin-1, a pro-resolving mediator, also depends upon interaction of neutrophils with platelets in a model of acute lung injury [150]. Indeed, the process of trans-metabolic production of pro-resolving mediators by platelet–leukocyte aggregates in inflammation is a burgeoning field, and the interested reader is directed to several excellent reviews of this area.

## Downstream consequences of platelet–leukocyte interactions for leukocyte function

The consequences of platelet binding for leukocyte function and fate have not been extensively investigated. However, acute modifications to leukocyte surface receptors are documented. Thus, in heterotypic aggregates, monocyte L-selectin is shed [151], while the expression and activity of β1- and β2-integrins is increased [65, 151–154]. Changes in the markers that define monocyte subsets are also altered by platelet binding. Thus, in a PGE2 driven process, CD16 on monocytes was increased in heterotypic aggregates formed *in vivo* after influenza vaccination or *in vitro* by mixing monocytes with platelets [155].

Leukocyte function after adhesion and activation on monolayers of activated platelets also changes. Thus, neutrophils activated on this substrate by IL-8, fMLP or C5a, released more neutrophil elastase than neutrophils in suspension. This process required β2-integrin (CD11b/CD18)-mediated binding to the platelets, and the production of LTB4 [33]. Neutrophils also receive signals from platelet adhesion receptors (P-selectin and CD31) which regulate the direction [32] and the velocity [31] of their migration. Platelet monolayers also supported the migration of adherent neutrophils into large, homotypic aggregates which were prone to detachment and dissemination downstream by flow, in a model of embolus formation [156]. The influence of platelet adhesion and/or exposure to platelet-derived agents such as chemokines has been reported to influence the function of a number of lymphocyte subsets, including helper T-cells, cytotoxic T-cells, regulatory T-cells, B-cells and NK cells. This area of lymphocyte biology has recently been reviewed and the reader is directed to the manuscript by Li et al. [65].

An aspect of leukocyte biology that has received surprisingly little attention is the role of platelet adhesion in the fate of cells as they differentiate. Scheuerer et al. [157] demonstrated that the platelet-derived chemokine, CXCL4, promoted the survival and differentiation of monocytes into macrophages. Interestingly, monocytes maturing in the presence of oxidised-low density lipoprotein (Ox-LDL) into foam cells also showed a CXCL4-dependent increase in esterification and storage of cholesterol [158]. Using microarray analysis, Gleissner et al. [159] showed acquisition of a ‘unique transcriptome’ in human monocyte-derived macrophages when they were differentiated in the presence of CXCL4. Interestingly, human macrophages differentiated in the presence of CXCL4 were unable to up-regulate athero-protective genes such as heam oxygenase-1, due to down regulation of the haemoglobin scavenger receptor, CD16 [3, 160]. In addition, binding of platelets to monocytes via P-selectin induced the secretion of TNF-α, IL-1β, IL-6, IL-12, CXCL8 and CCL4 (MIP-1β) [161]. Seta et al. [162] showed that in the presence of platelets or platelet-derived supernatants containing CXCL12 (SDF-1), monocytes could differentiate into monocyte-derived multipotential cells (MOMCs). These authors had previously described MOMCs as ‘primitive’ cells with the potential to differentiate into mesenchymal and endothelial lineages [163].

## The interaction between platelets and leukocytes as a therapeutic target in atherosclerosis

**Figure 3.**
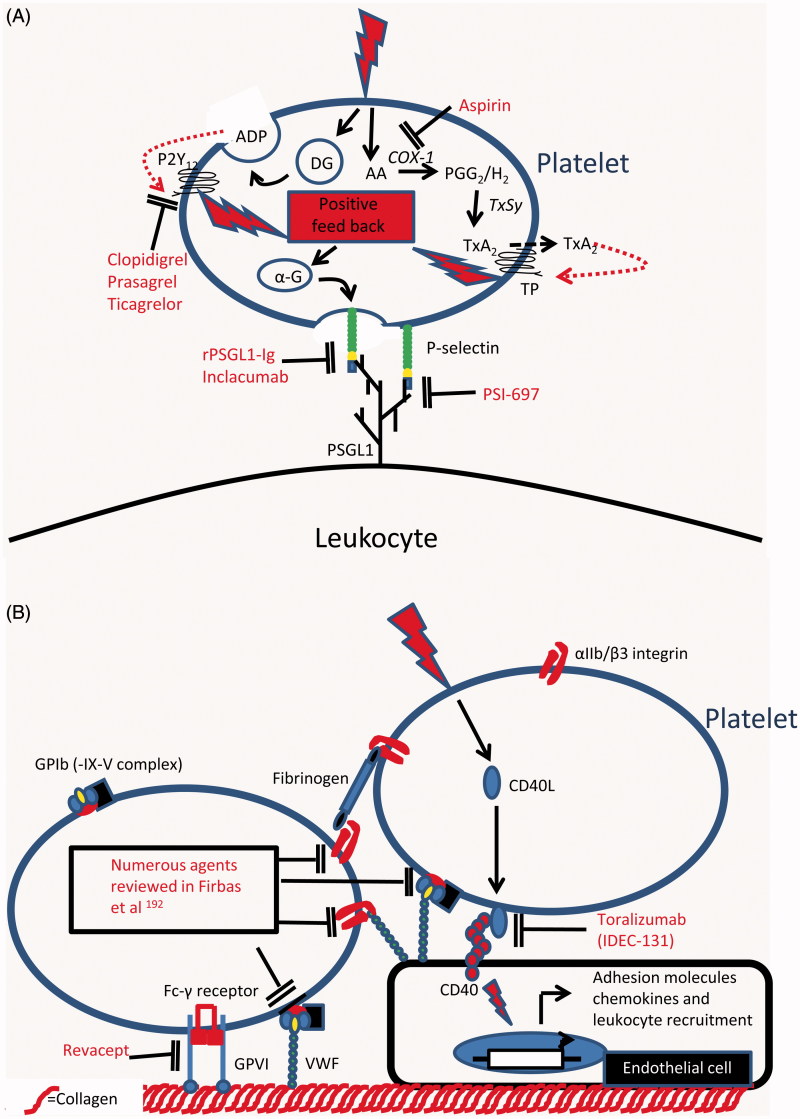
Targets for therapeutic intervention in platelet–leukocyte adhesion and in the platelet-mediated recruitment of leukocytes: (A) Therapeutic agents that inhibit the adhesive interactions between platelets and leukocytes or which inhibit the activation of platelets by antagonising platelet derived positive feedback loops which amplify the platelet response to primary activating stimuli. (B) Therapeutic agents that inhibit platelet adhesion to the vessel wall by either antagonising adhesive pathways directly or by inhibiting endothelial cell activation in response to platelet borne activating stimuli. AA, Arachidonic acid; ADP, Adenosine diphosphate; α-G, Alpha granule; COX-1, Cyclooxygenase-1; DG, Dense granule; P2Y12, ADP receptor; PGG2, Prostaglandin G2; PSGL1, P-selectin glycoprotein ligand1; TP, Thromboxane A2 receptor; TxA2, Thromboxane A2; TxSy, Thromboxane synthase.

As the preceding discussion has demonstrated, platelets can play a role in the recruitment of leukocytes during inflammation, and this pathway probably contributes to pathogenesis in atherosclerotic disease [69, 164]. Indeed, platelets increase the selective recruitment of monocytes to endothelium in animal and human models of vascular inflammation [45, 151, 164, 165]. In view of this and the positive association between platelets–leukocyte interaction and the progression of atherosclerosis, targeting these pathways represents a therapeutic avenue of potential importance. Figure 3 shows the pathways that are amenable to therapeutic intervention using currently licensed agents or drugs under development.

The anti-platelet agents currently used in prevention of athero-thrombotic disease target platelet activation in thrombotic events [166–168]. While the anti-thrombotic efficacy of guideline-approved agents such aspirin and P2Y12 receptor antagonists (clopidogrel, prasugrel and ticagrelor) is well established, recent evidence suggests they may have additional anti-inflammatory effects in addition to being platelet activation inhibitors [166–168]. Thus, Aspirin inhibits platelet activation by irreversibly binding to the platelet cyclooxygenase (COX)-1 enzyme, abolishing the formation of thromboxane (Tx)A2 [169]. Aspirin is ≈150 times more potent at acetylating ser529 on COX-1 than ser516 on its inducible homologue COX-2 [169]. Thus, at doses used for prevention of athero-thrombotic disease, aspirin has a negligible anti-inflammatory effect [166]. It is perhaps not surprising therefore that these doses of aspirin do not affect platelet or leukocyte activation and aggregate formation in whole blood [170].

Production of ADP by activated platelets amplifies platelet responses to activation, e.g. by stabilising platelet aggregation. ADP acts through the P2Y12 receptor [171]. Thus, the addition of a P2Y12 antagonist to aspirin offers better protection against athero-thrombotic ischemic events, especially in patients suffering from acute coronary syndromes or having undergone percutaneous coronary intervention [167]. There are currently three anti-platelet agents targeting this receptor: clopidogrel, and the newer generation prasugrel and ticagrelor [171]. In addition to potent antithrombotic efficacy, treatment with *clopidogrel* has been associated with a number of anti-inflammatory effects. In patients with atherosclerosis, clopidogrel caused a reduction in circulating CD40L and CCL-5, improved endothelial nitric oxide bioavailability, reduced platelet P-selectin expression and impaired formation platelet–leukocyte aggregates [172–177]. Interestingly, Antonino et al. showed that inhibition of inflammation markers correlated with inhibition of platelet activation, thus strengthening the hypothesis that clopidogrel’s anti-inflammatory effects are driven by inhibition of platelet activation [178]. *Prasugrel* is a P2Y12 receptor antagonist with more uniform and potent platelet inhibition than clopidogrel [171]. Consistent with this, prasugrel showed a greater reduction in heterotypic platelet–monocyte aggregates and levels of circulating CD40L [179–181]. Unlike clopidogrel and prasugrel, which require bioactivation by the liver, *ticagrelor* is a direct-acting reversible P2Y12 receptor antagonist [171]. In trials, ticagrelor was superior to clopidogrel in anti-thrombotic effects and in reduction of clinical endpoints of cardiovascular events and death [182]. Despite this, no differences in inflammatory markers was seen between clopidogrel- and ticagrelor-treated patients [183]. These studies imply that P2Y12 receptor antagonists have additional anti-inflammatory properties which go beyond inhibition of platelet activation.

Despite the anti-inflammatory effects seen with current anti-platelet agents, their use in secondary prevention of cardiovascular disease does not prevent athero-progression in humans [184]. This may be because their use is initiated late in disease development, often after an athero-thrombotic event. Alternatively, we may require a different approach to target this aspect of disease with greater precision, of which, inhibiting platelet-mediated leukocyte recruitment could be an important therapeutic endpoint. As platelet-facilitated leukocyte recruitment requires P-selectin interactions with PSGL-1 [185], inhibitors of this thrombo-inflammatory axis are in development. Among the earliest, a recombinant antagonist (rPSGL-Ig) was promising in pre-clinical studies. It significantly reduced the formation of heterotypic aggregates, attenuated infarct size, protected against IR injury and accelerated thrombolysis in animal models [186–190]. However, in clinical trials, the molecule showed no benefit to coronary vessel patency, infarct size or reperfusion and functional recovery of the myocardium. Indeed, clinical outcomes were unaltered at 1 or 6 months [191, 192].Consequently, development was stopped and ongoing studies terminated. Notwithstanding, novel P-selectin inhibitors are in development. A small molecule P-selectin antagonist, PSI-697, was shown to reduce *ex vivo* thrombus formation in humans, but failed to inhibit formation of heterotypic aggregates *in vivo* [71, 193]. Inclacumab, a human anti-P-selectin antibody, inhibited the formation of heterotypic aggregates in healthy volunteers and in patients with elevated numbers of circulating aggregates [72]. Whether these molecules will confer clinical benefit remains to be seen.

The CD40–CD40L interaction is an important link between platelet activation and inflammation [194]. Binding of platelet-released CD40L to CD40 on ECs, monocytes/macrophages, B cells and smooth muscle cells induces activatory changes that result in a pro-inflammatory phenotype [194]. Attempts have been made to block this interaction in order to halt athero-progression. In animal models, infusion of anti-CD40L antibodies delayed lesion formation, reduced leukocyte recruitment to the vessel wall and inhibited athero-progression [195–198]. A humanized antibody against CD40L (toralizumab, IDEC-131) was taken to phase II clinical trials in diseases with a strong inflammatory component (namely, multiple sclerosis and Crohn’s disease). Toralizumab’s development was stopped when a strong signal for unexplained risk of thrombosis emerged [199]. While the reason for thrombotic risk remains unexplained, development of compounds targeting the CD40L signalling pathway continues [194].

Intervention in platelet-mediated recruitment of leukocytes may also halt athero-progression. Platelet activation through the binding of the GPIb receptor to VWF contributes to leukocyte recruitment at high shear [200, 201, see above]. Moreover, blockade of platelet adhesion to arterial endothelium in atherosclerotic mice reduces the development of plaques as well as leukocyte recruitment to lesions [57 and see above]. Numerous inhibitors, antibodies, peptides and small molecules have been developed to target this interaction with promising pre-clinical results. As the prospects for therapies based on targeting this interaction have recently been discussed, the reader is directed to the review by Firbas et al. for further information [202]. Platelet adhesion at sites of vascular injury is an orchestrated event, in which the collagen receptor GPVI plays an important role [203]. Inhibition of GPVI protected animals from developing atherosclerotic lesions in mouse and rabbit models [204]. Revacept, a dimeric GPVI-Fc, favourably modified plaque morphology in atherosclerotic rabbits [205] and reduced cerebral infarct size and oedema in murine stroke [206]. This agent also inhibited collagen-induced responses in healthy volunteers, and was safe in a phase I trial [207]. Whether this molecule will have an impact on athero-progression in humans again remains unknown.

## Conclusions and future perspectives

There is now strong evidence that platelet–leukocyte interactions within the vasculature increase in cardiovascular disease, or even upon acquisition of risk factors for such diseases (e.g. hypertension or dyslipidaemia). A major question to be answered is whether these interactions are consequential or causal in inflammatory disease. Certainly, platelets are important in disease progression in animal models of atherosclerosis. Moreover, both *in vivo* and *in vitro* data suggests that part of their contribution to pathology is by recruitment of leukocytes to the vessel wall. Apparently direct interaction of platelets with arterial ECs can lead to this increase in leukocyte trafficking. Interestingly, there is sufficient evidence to suggest that this process selectivity recruits monocytes, which are the precursors of inflammatory foam cells in atherosclerotic plaque. Other modes of platelet leukocyte interaction that lead to leukocyte recruitment should not be discounted. The formation of heterotypic aggregates in circulating blood is known to increase in many pathologies, including cardiovascular diseases. However, whether these interactions are consequential or causal of disease remains to be determined experimentally. The developing field of microvesicle biology is likely to have an important influence on our understanding of platelet-mediated leukocyte recruitment in cardiovascular diseases. PMV play an important role in the thrombotic disease and coagulopathies [208] and they have been shown to interact with ECs *in vitro*, resulting in the recruitment of leukocytes. Like platelets, PMV form heterotypic aggregates with leukocytes in whole blood (unpublished observations). Again, whether these interactions are consequential or causal of disease remains to be determined experimentally.

So far, the known routes of platelet-mediated leukocyte recruitment are based on adhesion receptors and signalling pathways that regulate haemostasis and leukocyte trafficking during inflammation. However, it would be naïve to think that these represent an exhaustive list. Probably, new adhesive and regulatory pathways are still to be described. Indeed, the identification of CLEC-2 [209], a novel activation receptor on platelets is an exemplar of such a scenario. The only known physiological ligand for CLEC-2 is podoplanin [210], and Herzog et al. [211] showed that interactions between platelet borne CLEC-2 and podoplanin on the fibroblastic reticular cells cuffing high endothelial venules (HEV) of lymph nodes was necessary for integrity of this vascular bed and to ensure the regulated traffic of lymphocytes. Although this interaction maintains a homeostatic pathway in the immune system, it is likely that novel modes of platelet–leukocyte–vessel wall interaction will support pathological interactions in cardiovascular disease.

Whether intervening in platelet-mediated leukocyte recruitment will have efficacy in cardiovascular disease is still an open question. In animal models, blockade of platelet activation or platelet adhesion retards plaque development. However, in patients with established atherosclerosis, current anti-platelet therapies do not effect disease progression. Current therapeutic options were not designed to target platelets involved in these pathways and this could account for this lack of efficacy. As our understanding of the biology underlying these pathways increases, so should our ability to precisely target them with pharmacological agents.

## References

[CIT0001] Jackson SP (2011). Arterial thrombosis – Insidious, unpredictable and deadly. Nat Med.

[CIT0002] Hoebe K, Janssen E, Beutler B (2004). The interface between innate and adaptive immunity. Nat Immunol.

[CIT0003] Dunon D, Piali L, Imhof BA (1996). To stick or not to stick: The new leukocyte homing paradigm. Curr Opin Cell Biol.

[CIT0004] Liu Y, Shaw SK, Ma S, Yang L, Luscinskas FW, Parkos CA (2004). Regulation of leukocyte transmigration: Cell surface interactions and signaling events. J Immunol.

[CIT0005] Springer TA (1995). Traffic signals on endothelium for lymphocyte recirculation and leukocyte emigration. Annu Rev Physiol.

[CIT0006] Kansas GS (1996). Selectins and their ligands: Current concepts and controversies. Blood.

[CIT0007] Lawrence MB, Springer TA (1991). Leukocytes roll on a selectin at physiologic flow rates: Distinction from and prerequisite for adhesion through integrins. Cell.

[CIT0008] Takahashi M, Masuyama J, Ikeda U, Kitagawa S, Kasahara T, Saito M, Kano S, Shimada K (1995). Effects of endogenous endothelial interleukin-8 on neutrophil migration across an endothelial monolayer. Cardiovasc Res.

[CIT0009] Luu NT, Rainger GE, Nash GB (2000). Differential ability of exogenous chemotactic agents to disrupt transendothelial migration of flowing neutrophils. J Immunol.

[CIT0010] Rainger GE, Fisher AC, Nash GB (1997). Endothelial-borne platelet-activating factor and interleukin-8 rapidly immobilize rolling neutrophils. Am J Physiol.

[CIT0011] Belanger C, Elimam H, Lefebvre J, Borgeat P, Marleau S (2008). Involvement of endogenous leukotriene b4 and platelet-activating factor in polymorphonuclear leucocyte recruitment to dermal inflammatory sites in rats. Immunology.

[CIT0012] Kim ND, Chou RC, Seung E, Tager AM, Luster AD (2006). A unique requirement for the leukotriene b4 receptor blt1 for neutrophil recruitment in inflammatory arthritis. J Exp Med.

[CIT0013] Prescott SM, Zimmerman GA, Stafforini DM, McIntyre TM (2000). Platelet-activating factor and related lipid mediators. Annu Rev Biochem.

[CIT0014] Rollin S, Lemieux C, Maliba R, Favier J, Villeneuve LR, Allen BG, Soker S, Bazan NG, Merhi Y, Sirois MG (2004). Vegf-mediated endothelial p-selectin translocation: Role of vegf receptors and endogenous paf synthesis. Blood.

[CIT0015] Ahmed SR, McGettrick HM, Yates CM, Buckley CD, Ratcliffe MJ, Nash GB, Rainger GE (2011). Prostaglandin D2 regulates CD4+ memory T cell trafficking across blood vascular endothelium and primes these cells for clearance across lymphatic endothelium. J Immunol.

[CIT0016] Muller WA (1995). The role of pecam-1 (cd31) in leukocyte emigration: Studies *in vitro* and *in vivo*. J Leukoc Biol.

[CIT0017] Muller WA (2009). Mechanisms of transendothelial migration of leukocytes. Circ Res.

[CIT0018] Serhan CN, Brain SD, Buckley CD, Gilroy DW, Haslett C, O'Neill LA, Perretti M, Rossi AG, Wallace JL (2007). Resolution of inflammation: State of the art, definitions and terms. FASEB J.

[CIT0019] Hamburger SA, McEver RP (1990). Gmp-140 mediates adhesion of stimulated platelets to neutrophils. Blood.

[CIT0020] Larsen E, Celi A, Gilbert GE, Furie BC, Erban JK, Bonfanti R, Wagner DD, Furie B (1989). Padgem protein: A receptor that mediates the interaction of activated platelets with neutrophils and monocytes. Cell.

[CIT0021] Buttrum SM, Hatton R, Nash GB (1993). Selectin-mediated rolling of neutrophils on immobilized platelets. Blood.

[CIT0022] Lalor P, Nash GB (1995). Adhesion of flowing leucocytes to immobilized platelets. Br J Haematol.

[CIT0023] Stone PC, Nash GB (1999). Conditions under which immobilized platelets activate as well as capture flowing neutrophils. Br J Haematol.

[CIT0024] Weber C, Springer TA (1997). Neutrophil accumulation on activated, surface-adherent platelets in flow is mediated by interaction of mac-1 with fibrinogen bound to alphaiibbeta3 and stimulated by platelet-activating factor. J Clin Invest.

[CIT0025] Ostrovsky L, King AJ, Bond S, Mitchell D, Lorant DE, Zimmerman GA, Larsen R, Niu XF, Kubes P (1998). A juxtacrine mechanism for neutrophil adhesion on platelets involves platelet-activating factor and a selectin-dependent activation process. Blood.

[CIT0026] Antoine C, Murphy RC, Henson PM, Maclouf J (1992). Time-dependent utilization of platelet arachidonic acid by the neutrophil in formation of 5-lipoxygenase products in platelet-neutrophil co-incubations. Biochim Biophys Acta.

[CIT0027] Power CA, Furness RB, Brawand C, Wells TN (1994). Cloning of a full-length cDNA encoding the neutrophil-activating peptide ena-78 from human platelets. Gene.

[CIT0028] Su SB, Mukaida N, Matsushima K (1996). Rapid secretion of intracellularly pre-stored interleukin-8 from rabbit platelets upon activation. J Leukoc Biol.

[CIT0029] Diacovo TG, Roth SJ, Buccola JM, Bainton DF, Springer TA (1996). Neutrophil rolling, arrest, and transmigration across activated, surface-adherent platelets via sequential action of p-selectin and the beta 2-integrin cd11b/cd18. Blood.

[CIT0030] Sheikh S, Nash GB (1996). Continuous activation and deactivation of integrin cd11b/cd18 during de novo expression enables rolling neutrophils to immobilize on platelets. Blood.

[CIT0031] Rainger GE, Buckley C, Simmons DL, Nash GB (1997). Cross-talk between cell adhesion molecules regulates the migration velocity of neutrophils. Curr Biol.

[CIT0032] Rainger GE, Buckley CD, Simmons DL, Nash GB (1999). Neutrophils sense flow-generated stress and direct their migration through alphavbeta3-integrin. Am J Physiol.

[CIT0033] Rainger GE, Buckley C, Simmons DL, Nash GB (1998). Neutrophils rolling on immobilised platelets migrate into homotypic aggregates after activation. Thromb Haemost.

[CIT0034] Ware JA, Heistad DD (1993). Seminars in medicine of the beth israel hospital, boston. Platelet-endothelium interactions. N Engl J Med.

[CIT0035] Radomski MW, Palmer RM, Moncada S (1987). Endogenous nitric oxide inhibits human platelet adhesion to vascular endothelium. Lancet.

[CIT0036] Radomski MW, Palmer RM, Moncada S (1987). The anti-aggregating properties of vascular endothelium: Interactions between prostacyclin and nitric oxide. Br J Pharmacol.

[CIT0037] Radomski MW, Palmer RM, Moncada S (1991). Modulation of platelet aggregation by an l-arginine-nitric oxide pathway. Trends Pharmacol Sci.

[CIT0038] Davies MJ, Woolf N, Rowles PM, Pepper J (1988). Morphology of the endothelium over atherosclerotic plaques in human coronary arteries. Br Heart J.

[CIT0039] Rosenblum WI (1997). Platelet adhesion and aggregation without endothelial denudation or exposure of basal lamina and/or collagen. J Vasc Res.

[CIT0040] Kuijper PH, Gallardo Tores HI, Lammers JW, Sixma JJ, Koenderman L, Zwaginga JJ (1998). Platelet associated fibrinogen and icam-2 induce firm adhesion of neutrophils under flow conditions. Thromb Haemost.

[CIT0041] Bombeli T, Schwartz BR, Harlan JM (1998). Adhesion of activated platelets to endothelial cells: Evidence for a gpiibiiia-dependent bridging mechanism and novel roles for endothelial intercellular adhesion molecule 1 (icam-1), alphavbeta3 integrin, and gpibalpha. J Exp Med.

[CIT0042] Gawaz M, Neumann FJ, Dickfeld T, Reininger A, Adelsberger H, Gebhardt A, Schomig A (1997). Vitronectin receptor (alpha(v)beta3) mediates platelet adhesion to the luminal aspect of endothelial cells: Implications for reperfusion in acute myocardial infarction. Circulation.

[CIT0043] Kaplan JE, Moon DG, Weston LK, Minnear FL, Del Vecchio PJ, Shepard JM, Fenton JW (1989). Platelets adhere to thrombin-treated endothelial cells in vitro. Am J Physiol.

[CIT0044] Kirton CM, Nash GB (2000). Activated platelets adherent to an intact endothelial cell monolayer bind flowing neutrophils and enable them to transfer to the endothelial surface. J Lab Clin Med.

[CIT0045] Kuckleburg CJ, Yates CM, Kalia N, Zhao Y, Nash GB, Watson SP, Rainger GE (2011). Endothelial cell-borne platelet bridges selectively recruit monocytes in human and mouse models of vascular inflammation. Cardiovasc Res.

[CIT0046] Lagadec P, Dejoux O, Ticchioni M, Cottrez F, Johansen M, Brown EJ, Bernard A (2003). Involvement of a cd47-dependent pathway in platelet adhesion on inflamed vascular endothelium under flow. Blood.

[CIT0047] Radomski MW, Vallance P, Whitley G, Foxwell N, Moncada S (1993). Platelet adhesion to human vascular endothelium is modulated by constitutive and cytokine induced nitric oxide. Cardiovasc Res.

[CIT0048] Reininger AJ, Agneskirchner J, Bode PA, Spannagl M, Wurzinger LJ (2000). C7e3 fab inhibits low shear flow modulated platelet adhesion to endothelium and surface-absorbed fibrinogen by blocking platelet gp iib/iiia as well as endothelial vitronectin receptor – Results from patients with acute myocardial infarction and healthy controls. Thromb Haemost.

[CIT0049] Reininger AJ, Korndorfer MA, Wurzinger LJ (1998). Adhesion of adp-activated platelets to intact endothelium under stagnation point flow *in vitro* is mediated by the integrin alphaiibeta3. Thromb Haemost.

[CIT0050] Rosenblum WI, Nelson GH, Wormley B, Werner P, Wang J, Shih CC (1996). Role of platelet-endothelial cell adhesion molecule (pecam) in platelet adhesion/aggregation over injured but not denuded endothelium *in vivo* and *ex vivo*. Stroke.

[CIT0051] Shanberge JN, Kajiwara Y, Quattrociocchi-Longe T (1995). Effect of aspirin and iloprost on adhesion of platelets to intact endothelium *in vivo*. J Lab Clin Med.

[CIT0052] Tomita Y, Tanahashi N, Tomita M, Itoh Y, Yokoyama M, Takeda H, Schiszler I, Fukuuchi Y (2001). Role of platelet glycoprotein iib/iiia in adp-activated platelet adhesion to aortic endothelial cells *in vitro*: Observation with video-enhanced contrast microscopy. Clin Hemorheol Microcirc.

[CIT0053] Tull SP, Anderson SI, Hughan SC, Watson SP, Nash GB, Rainger GE (2006). Cellular pathology of atherosclerosis: Smooth muscle cells promote adhesion of platelets to cocultured endothelial cells. Circ Res.

[CIT0054] Venturini CM, Weston LK, Kaplan JE (1992). Platelet cgmp, but not camp, inhibits thrombin-induced platelet adhesion to pulmonary vascular endothelium. Am J Physiol.

[CIT0055] Andre P, Denis CV, Ware J, Saffaripour S, Hynes RO, Ruggeri ZM, Wagner DD (2000). Platelets adhere to and translocate on von willebrand factor presented by endothelium in stimulated veins. Blood.

[CIT0056] Huo Y, Schober A, Forlow SB, Smith DF, Hyman MC, Jung S, Littman DR, Weber C, Ley K (2003). Circulating activated platelets exacerbate atherosclerosis in mice deficient in apolipoprotein e. Nat Med.

[CIT0057] Massberg S, Brand K, Gruner S, Page S, Muller E, Muller I, Bergmeier W, Richter T, Lorenz M, Konrad I, Nieswandt B, Gawaz M (2002). A critical role of platelet adhesion in the initiation of atherosclerotic lesion formation. J Exp Med.

[CIT0058] Frenette PS, Johnson RC, Hynes RO, Wagner DD (1995). Platelets roll on stimulated endothelium *in vivo*: An interaction mediated by endothelial p-selectin. Proc Natl Acad Sci USA.

[CIT0059] van Gils JM, da Costa Martins PA, Mol A, Hordijk PL, Zwaginga JJ (2008). Transendothelial migration drives dissociation of plateletmonocyte complexes. Thromb Haemost.

[CIT0060] Kuijper PH, Gallardo Torres HI, van der Linden JA, Lammers JW, Sixma JJ, Koenderman L, Zwaginga JJ (1996). Platelet-dependent primary hemostasis promotes selectin- and integrin-mediated neutrophil adhesion to damaged endothelium under flow conditions. Blood.

[CIT0061] Bahra P, Nash GB (1998). Sparsely adherent platelets support capture and immobilization of flowing neutrophils. J Lab Clin Med.

[CIT0062] Butler LM, Metson-Scott T, Felix J, Abhyankar A, Rainger GE, Farndale RW, Watson SP, Nash GB (2007). Sequential adhesion of platelets and leukocytes from flowing whole blood onto a collagen-coated surface: Requirement for a gpvi-binding site in collagen. Thromb Haemost.

[CIT0063] Skilbeck CA, Walker PG, David T, Nash GB (2004). Disturbed flow promotes deposition of leucocytes from flowing whole blood in a model of a damaged vessel wall. Br J Haematol.

[CIT0064] da Costa Martins P, van den Berk N, Ulfman LH, Koenderman L, Hordijk PL, Zwaginga JJ (2004). Platelet-monocyte complexes support monocyte adhesion to endothelium by enhancing secondary tethering and cluster formation. Arterioscler Thromb Vasc Biol.

[CIT0065] Li N (2008). Platelet-lymphocyte cross-talk. J Leukoc Biol.

[CIT0066] Zarbock A, Polanowska-Grabowska RK, Ley K (2007). Platelet-neutrophil-interactions: Linking hemostasis and inflammation. Blood Rev.

[CIT0067] von Hundelshausen P, Weber KS, Huo Y, Proudfoot AE, Nelson PJ, Ley K, Weber C (2001). Rantes deposition by platelets triggers monocyte arrest on inflamed and atherosclerotic endothelium. Circulation.

[CIT0068] Schulz C, Schafer A, Stolla M, Kerstan S, Lorenz M, von Bruhl ML, Schiemann M, Bauersachs J, Gloe T, Busch DH (2007). Chemokine fractalkine mediates leukocyte recruitment to inflammatory endothelial cells in flowing whole blood: A critical role for p-selectin expressed on activated platelets. Circulation.

[CIT0069] van Gils JM, Zwaginga JJ, Hordijk PL (2009). Molecular and functional interactions among monocytes, platelets, and endothelial cells and their relevance for cardiovascular diseases. J Leukoc Biol.

[CIT0070] Rinder HM, Bonan JL, Rinder CS, Ault KA, Smith BR (1991). Dynamics of leukocyte-platelet adhesion in whole blood. Blood.

[CIT0071] Japp AG, Chelliah R, Tattersall L, Lang NN, Meng X, Weisel K, Katz A, Burt D, Fox KA, Feuerstein GZ (2013). Effect of psi-697, a novel p-selectin inhibitor, on platelet-monocyte aggregate formation in humans. J Am Heart Assoc.

[CIT0072] Kling D, Stucki C, Kronenberg S, Tuerck D, Rheaume E, Tardif JC, Gaudreault J, Schmitt C (2013). Pharmacological control of platelet-leukocyte interactions by the human anti-p-selectin antibody inclacumab – Preclinical and clinical studies. Thromb Res.

[CIT0073] Fernandes LS, Conde ID, Wayne Smith C, Kansas GS, Snapp KR, Bennet N, Ballantyne C, McIntire LV, O'Brian Smith E, Klem JA (2003). Platelet-monocyte complex formation: Effect of blocking psgl-1 alone, and in combination with alphaiibbeta3 and alphambeta2, in coronary stenting. Thromb Res.

[CIT0074] Simon DI, Chen Z, Xu H, Li CQ, Dong J, McIntire LV, Ballantyne CM, Zhang L, Furman MI, Berndt MC (2000). Platelet glycoprotein ibalpha is a counterreceptor for the leukocyte integrin mac-1 (cd11b/cd18). J Exp Med.

[CIT0075] Yip C, Ignjatovic V, Attard C, Monagle P, Linden MD (2013). First report of elevated monocyte-platelet aggregates in healthy children. PLoS One.

[CIT0076] Elalamy I, Chakroun T, Gerotziafas GT, Petropoulou A, Robert F, Karroum A, Elgrably F, Samama MM, Hatmi M (2008). Circulating platelet-leukocyte aggregates: A marker of microvascular injury in diabetic patients. Thromb Res.

[CIT0077] Harding SA, Sommerfield AJ, Sarma J, Twomey PJ, Newby DE, Frier BM, Fox KA (2004). Increased cd40 ligand and platelet-monocyte aggregates in patients with type 1 diabetes mellitus. Atherosclerosis.

[CIT0078] Joseph JE, Harrison P, Mackie IJ, Isenberg DA, Machin SJ (2001). Increased circulating platelet-leucocyte complexes and platelet activation in patients with antiphospholipid syndrome, systemic lupus erythematosus and rheumatoid arthritis. Br J Haematol.

[CIT0079] Tekelioglu Y, Uzun H, Gucer H (2013). Circulating platelet-leukocyte aggregates in patients with inflammatory bowel disease. J Chin Med Assoc.

[CIT0080] Wrigley BJ, Shantsila E, Tapp LD, Lip GY (2013). Increased formation of monocyte-platelet aggregates in ischemic heart failure. Circ Heart Fail.

[CIT0081] Goncalves R, Zhang X, Cohen H, Debrabant A, Mosser DM (2011). Platelet activation attracts a subpopulation of effector monocytes to sites of leishmania major infection. J Exp Med.

[CIT0082] Parimon T, Li Z, Bolz DD, McIndoo ER, Bayer CR, Stevens DL, Bryant AE (2013). *Staphylococcus aureus* alpha-hemolysin promotes platelet-neutrophil aggregate formation. J Infect Dis.

[CIT0083] Furman MI, Barnard MR, Krueger LA, Fox ML, Shilale EA, Lessard DM, Marchese P, Frelinger AL, Goldberg RJ, Michelson AD (2001). Circulating monocyte-platelet aggregates are an early marker of acute myocardial infarction. J Am Coll Cardiol.

[CIT0084] Furman MI, Benoit SE, Barnard MR, Valeri CR, Borbone ML, Becker RC, Hechtman HB, Michelson AD (1998). Increased platelet reactivity and circulating monocyte-platelet aggregates in patients with stable coronary artery disease. J Am Coll Cardiol.

[CIT0085] Htun P, Fateh-Moghadam S, Tomandl B, Handschu R, Klinger K, Stellos K, Garlichs C, Daniel W, Gawaz M (2006). Course of platelet activation and platelet-leukocyte interaction in cerebrovascular ischemia. Stroke.

[CIT0086] McCabe DJ, Harrison P, Mackie IJ, Sidhu PS, Purdy G, Lawrie AS, Watt H, Brown MM, Machin SJ (2004). Platelet degranulation and monocyte-platelet complex formation are increased in the acute and convalescent phases after ischaemic stroke or transient ischaemic attack. Br J Haematol.

[CIT0087] Michelson AD, Barnard MR, Krueger LA, Valeri CR, Furman MI (2001). Circulating monocyte-platelet aggregates are a more sensitive marker of *in vivo* platelet activation than platelet surface p-selectin: Studies in baboons, human coronary intervention, and human acute myocardial infarction. Circulation.

[CIT0088] Mickelson JK, Lakkis NM, Villarreal-Levy G, Hughes BJ, Smith CW (1996). Leukocyte activation with platelet adhesion after coronary angioplasty: A mechanism for recurrent disease?. J Am Coll Cardiol.

[CIT0089] Smout J, Dyker A, Cleanthis M, Ford G, Kesteven P, Stansby G (2009). Platelet function following acute cerebral ischemia. Angiology.

[CIT0090] Gkaliagkousi E, Corrigall V, Becker S, de Winter P, Shah A, Zamboulis C, Ritter J, Ferro A (2009). Decreased platelet nitric oxide contributes to increased circulating monocyte-platelet aggregates in hypertension. Eur Heart J.

[CIT0091] Nomura S, Kanazawa S, Fukuhara S (2002). Effects of efonidipine on platelet and monocyte activation markers in hypertensive patients with and without type 2 diabetes mellitus. J Hum Hypertens.

[CIT0092] Lippi G, Montagnana M, Salvagno GL, Cicorella N, Degan M, Minuz P, Lechi C, Guidi GC (2007). Risk stratification of patients with acute myocardial infarction by quantification of circulating monocyte-platelet aggregates. Int J Cardiol.

[CIT0093] Raposo G, Stoorvogel W (2013). Extracellular vesicles: Exosomes, microvesicles, and friends. J Cell Biol.

[CIT0094] Varon D, Hayon Y, Dashevsky O, Shai E (2012). Involvement of platelet derived microparticles in tumor metastasis and tissue regeneration. Thromb Res.

[CIT0095] Barteneva NS, Fasler-Kan E, Bernimoulin M, Stern JN, Ponomarev ED, Duckett L, Vorobjev IA (2013). Circulating microparticles: Square the circle. BMC Cell Biol.

[CIT0096] Del Conde I, Shrimpton CN, Thiagarajan P, Lopez JA (2005). Tissue-factor-bearing microvesicles arise from lipid rafts and fuse with activated platelets to initiate coagulation. Blood.

[CIT0097] Yang C, Xiong W, Qiu Q, Shao Z, Hamel D, Tahiri H, Leclair G, Lachapelle P, Chemtob S, Hardy P (2012). Role of receptor-mediated endocytosis in the antiangiogenic effects of human t lymphoblastic cell-derived microparticles. Am J Physiol Regul Integr Comp Physiol.

[CIT0098] Orozco AF, Jorgez CJ, Horne C, Marquez-Do DA, Chapman MR, Rodgers JR, Bischoff FZ, Lewis DE (2008). Membrane protected apoptotic trophoblast microparticles contain nucleic acids: Relevance to preeclampsia. Am J Pathol.

[CIT0099] Risitano A, Beaulieu LM, Vitseva O, Freedman JE (2012). Platelets and platelet-like particles mediate intercellular RNA transfer. Blood.

[CIT0100] Hunter MP, Ismail N, Zhang X, Aguda BD, Lee EJ, Yu L, Xiao T, Schafer J, Lee ML, Schmittgen TD (2008). Detection of microrna expression in human peripheral blood microvesicles. PLoS One.

[CIT0101] Barry OP, Pratico D, Lawson JA, FitzGerald GA (1997). Transcellular activation of platelets and endothelial cells by bioactive lipids in platelet microparticles. J Clin Invest.

[CIT0102] Garcia BA, Smalley DM, Cho H, Shabanowitz J, Ley K, Hunt DF (2005). The platelet microparticle proteome. J Proteome Res.

[CIT0103] Flaumenhaft R, Dilks JR, Richardson J, Alden E, Patel-Hett SR, Battinelli E, Klement GL, Sola-Visner M, Italiano JE (2009). Megakaryocyte-derived microparticles: Direct visualization and distinction from platelet-derived microparticles. Blood.

[CIT0104] Cloutier N, Tan S, Boudreau LH, Cramb C, Subbaiah R, Lahey L, Albert A, Shnayder R, Gobezie R, Nigrovic PA (2013). The exposure of autoantigens by microparticles underlies the formation of potent inflammatory components: The microparticle-associated immune complexes. EMBO Mol Med.

[CIT0105] Holme PA, Rosger M, Solum NO, Brosstad F, Larsen AM, Hovig T (1996). Glycoprotein iib-iiia on platelet-derived microparticles, and microparticle structures studied by electron microscopy, confocal laser microscopy and crossed radio-immunoelectrophoresis. Platelets.

[CIT0106] Nomura S, Tandon NN, Nakamura T, Cone J, Fukuhara S, Kambayashi J (2001). High-shear-stress-induced activation of platelets and microparticles enhances expression of cell adhesion molecules in thp-1 and endothelial cells. Atherosclerosis.

[CIT0107] Shai E, Rosa I, Parguina AF, Motahedeh S, Varon D, Garcia A (2012). Comparative analysis of platelet-derived microparticles reveals differences in their amount and proteome depending on the platelet stimulus. J Proteomics.

[CIT0108] Ogata N, Imaizumi M, Nomura S, Shozu A, Arichi M, Matsuoka M, Matsumura M (2005). Increased levels of platelet-derived microparticles in patients with diabetic retinopathy. Diabetes Res Clin Pract.

[CIT0109] Nomura S, Suzuki M, Katsura K, Xie GL, Miyazaki Y, Miyake T, Kido H, Kagawa H, Fukuhara S (1995). Platelet-derived microparticles may influence the development of atherosclerosis in diabetes mellitus. Atherosclerosis.

[CIT0110] Feng B, Chen Y, Luo Y, Chen M, Li X, Ni Y (2010). Circulating level of microparticles and their correlation with arterial elasticity and endothelium-dependent dilation in patients with type 2 diabetes mellitus. Atherosclerosis.

[CIT0111] Biasucci LM, Porto I, Di Vito L, De Maria GL, Leone AM, Tinelli G, Tritarelli A, Di Rocco G, Snider F, Capogrossi MC (2012). Differences in microparticle release in patients with acute coronary syndrome and stable angina. Circ J.

[CIT0112] Tersteeg C, Heijnen HF, Eckly A, Pasterkamp G, Urbanus RT, Maas C, Hoefer IE, Nieuwland R, Farndale RW, Gachet C (2014). Flow-induced protrusions (FLIPRs): A platelet-derived platform for the retrieval of microparticles by monocytes and neutrophils. Circ Res.

[CIT0113] Mause SF, von Hundelshausen P, Zernecke A, Koenen RR, Weber C (2005). Platelet microparticles: A transcellular delivery system for rantes promoting monocyte recruitment on endothelium. Arterioscler Thromb Vasc Biol.

[CIT0114] Barry OP, FitzGerald GA (1999). Mechanisms of cellular activation by platelet microparticles. Thromb Haemost.

[CIT0115] Barry OP, Pratico D, Savani RC, FitzGerald GA (1998). Modulation of monocyte-endothelial cell interactions by platelet microparticles. J Clin Invest.

[CIT0116] Lindemann S, Tolley ND, Dixon DA, McIntyre TM, Prescott SM, Zimmerman GA, Weyrich AS (2001). Activated platelets mediate inflammatory signaling by regulated interleukin 1beta synthesis. J Cell Biol.

[CIT0117] Forlow SB, McEver RP, Nollert MU (2000). Leukocyte-leukocyte interactions mediated by platelet microparticles under flow. Blood.

[CIT0118] Diepenhorst GM, van Gulik TM, Hack CE (2009). Complement-mediated ischemia-reperfusion injury: Lessons learned from animal and clinical studies. Ann Surg.

[CIT0119] Harris AG, Leiderer R, Peer F, Messmer K (1996). Skeletal muscle microvascular and tissue injury after varying durations of ischemia. Am J Physiol.

[CIT0120] Lehr HA, Menger MD, Messmer K (1993). Impact of leukocyte adhesion on myocardial ischemia/reperfusion injury: Conceivable mechanisms and proven facts. J Lab Clin Med.

[CIT0121] Ley K (1996). Molecular mechanisms of leukocyte recruitment in the inflammatory process. Cardiovasc Res.

[CIT0122] Nolte D, Hecht R, Schmid P, Botzlar A, Menger MD, Neumueller C, Sinowatz F, Vestweber D, Messmer K (1994). Role of mac-1 and icam-1 in ischemia-reperfusion injury in a microcirculation model of balb/c mice. Am J Physiol.

[CIT0123] Cywes R, Mullen JB, Stratis MA, Greig PD, Levy GA, Harvey PR, Strasberg SM (1993). Prediction of the outcome of transplantation in man by platelet adherence in donor liver allografts. Evidence of the importance of prepreservation injury. Transplantation.

[CIT0124] Cywes R, Packham MA, Tietze L, Sanabria JR, Harvey PR, Phillips MJ, Strasberg SM (1993). Role of platelets in hepatic allograft preservation injury in the rat. Hepatology.

[CIT0125] Flores NA, Goulielmos NV, Seghatchian MJ, Sheridan DJ (1994). Myocardial ischaemia induces platelet activation with adverse electrophysiological and arrhythmogenic effects. Cardiovasc Res.

[CIT0126] Okada Y, Marchevsky AM, Zuo XJ, Pass JA, Kass RM, Matloff JM, Jordan SC (1997). Accumulation of platelets in rat syngeneic lung transplants: A potential factor responsible for preservation-reperfusion injury. Transplantation.

[CIT0127] Khandoga A, Biberthaler P, Messmer K, Krombach F (2003). Platelet-endothelial cell interactions during hepatic ischemia-reperfusion *in vivo*: A systematic analysis. Microvasc Res.

[CIT0128] Kuroda T, Shiohara E, Homma T, Furukawa Y, Chiba S (1994). Effects of leukocyte and platelet depletion on ischemia – Reperfusion injury to dog pancreas. Gastroenterology.

[CIT0129] Chintala MS, Bernardino V, Chiu PJ (1994). Cyclic gmp but not cyclic amp prevents renal platelet accumulation after ischemia-reperfusion in anesthetized rats. J Pharmacol Exp Ther.

[CIT0130] Massberg S, Enders G, Leiderer R, Eisenmenger S, Vestweber D, Krombach F, Messmer K (1998). Platelet-endothelial cell interactions during ischemia/reperfusion: The role of p-selectin. Blood.

[CIT0131] Khandoga A, Biberthaler P, Enders G, Axmann S, Hutter J, Messmer K, Krombach F (2002). Platelet adhesion mediated by fibrinogen-intercelllular adhesion molecule-1 binding induces tissue injury in the postischemic liver *in vivo*. Transplantation.

[CIT0132] Khandoga A, Biberthaler P, Enders G, Teupser D, Axmann S, Luchting B, Hutter J, Messmer K, Krombach F (2002). P-selectin mediates platelet-endothelial cell interactions and reperfusion injury in the mouse liver *in vivo*. Shock.

[CIT0133] Cooper D, Chitman KD, Williams MC, Granger DN (2003). Time-dependent platelet-vessel wall interactions induced by intestinal ischemia-reperfusion. Am J Physiol Gastrointest Liver Physiol.

[CIT0134] Nishijima K, Kiryu J, Tsujikawa A, Honjo M, Nonaka A, Yamashiro K, Tanihara H, Tojo SJ, Ogura Y, Honda Y (2001). *In vivo* evaluation of platelet – Endothelial interactions after transient retinal ischemia. Invest Ophthalmol Vis Sci.

[CIT0135] Russell J, Cooper D, Tailor A, Stokes KY, Granger DN (2003). Low venular shear rates promote leukocyte-dependent recruitment of adherent platelets. Am J Physiol Gastrointest Liver Physiol.

[CIT0136] Yadav SS, Howell DN, Steeber DA, Harland RC, Tedder TF, Clavien PA (1999). P-selectin mediates reperfusion injury through neutrophil and platelet sequestration in the warm ischemic mouse liver. Hepatology.

[CIT0137] Campbell B, Chuhran CM, Lefer DJ, Lefer AM (1999). Cardioprotective effects of abciximab (reopro) in an isolated perfused rat heart model of ischemia and reperfusion. Methods Find Exp Clin Pharmacol.

[CIT0138] Kupatt C, Wichels R, Horstkotte J, Krombach F, Habazettl H, Boekstegers P (2002). Molecular mechanisms of platelet-mediated leukocyte recruitment during myocardial reperfusion. J Leukoc Biol.

[CIT0139] Massberg S, Enders G, Matos FC, Tomic LI, Leiderer R, Eisenmenger S, Messmer K, Krombach F (1999). Fibrinogen deposition at the postischemic vessel wall promotes platelet adhesion during ischemia-reperfusion *in vivo*. Blood.

[CIT0140] Ovechkin AV, Lominadze D, Sedoris KC, Gozal E, Robinson TW, Roberts AM (2005). Inhibition of inducible nitric oxide synthase attenuates platelet adhesion in subpleural arterioles caused by lung ischemia-reperfusion in rabbits. J Appl Physiol (1985).

[CIT0141] Ishikawa M, Sekizuka E, Yamaguchi N, Nakadate H, Terao S, Granger DN, Minamitani H (2007). Angiotensin ii type 1 receptor signaling contributes to platelet-leukocyte-endothelial cell interactions in the cerebral microvasculature. Am J Physiol Heart Circ Physiol.

[CIT0142] Uhlmann D, Glasser S, Gaebel G, Armann B, Ludwig S, Tannapfel A, Hauss J, Witzigmann H (2005). Improvement of postischemic hepatic microcirculation after endothelina receptor blockade – Endothelin antagonism influences platelet-endothelium interactions. J Gastrointest Surg.

[CIT0143] Osman M, Russell J, Granger DN (2009). Lymphocyte-derived interferon-gamma mediates ischemia-reperfusion-induced leukocyte and platelet adhesion in intestinal microcirculation. Am J Physiol Gastrointest Liver Physiol.

[CIT0144] Lefer AM, Campbell B, Scalia R, Lefer DJ (1998). Synergism between platelets and neutrophils in provoking cardiac dysfunction after ischemia and reperfusion: Role of selectins. Circulation.

[CIT0145] Salter JW, Krieglstein CF, Issekutz AC, Granger DN (2001). Platelets modulate ischemia/reperfusion-induced leukocyte recruitment in the mesenteric circulation. Am J Physiol Gastrointest Liver Physiol.

[CIT0146] Cooper D, Russell J, Chitman KD, Williams MC, Wolf RE, Granger DN (2004). Leukocyte dependence of platelet adhesion in postcapillary venules. Am J Physiol Heart Circ Physiol.

[CIT0147] Kuroda T, Shiohara E (1996). Leukocyte and platelet depletion protects the liver from damage induced by cholestasis and ischemia-reperfusion in the dog. Scand J Gastroenterol.

[CIT0148] Singbartl K, Forlow SB, Ley K (2001). Platelet, but not endothelial, p-selectin is critical for neutrophil-mediated acute postischemic renal failure. FASEB J.

[CIT0149] Brancaleone V, Gobbetti T, Cenac N, le Faouder P, Colom B, Flower RJ, Vergnolle N, Nourshargh S, Perretti M (2013). A vasculo-protective circuit centered on lipoxin A_4_ and aspirin-triggered 15-epi-lipoxin A_4_ operative in murine microcirculation. Blood.

[CIT0150] Abdulnour RE, Dalli J, Colby JK, Krishnamoorthy N, Timmons JY, Hwa Tan S, Colas RA, Petasis NA, Serhan CN, Levy BD (2014). Maresin 1 biosynthesis during platelet–neutrophil interactions is organ-protective. Proc Natl Acad Sci USA.

[CIT0151] Yago T, Tsukuda M, Minami M (1999). P-selectin binding promotes the adhesion of monocytes to vcam-1 under flow conditions. J Immunol.

[CIT0152] Bouchon A, Dietrich J, Colonna M (2000). Cutting edge: Inflammatory responses can be triggered by trem-1, a novel receptor expressed on neutrophils and monocytes. J Immunol.

[CIT0153] da Costa Martins PA, van Gils JM, Mol A, Hordijk PL, Zwaginga JJ (2006). Platelet binding to monocytes increases the adhesive properties of monocytes by up-regulating the expression and functionality of beta1 and beta2 integrins. J Leukoc Biol.

[CIT0154] Neumann FJ, Marx N, Gawaz M, Brand K, Ott I, Rokitta C, Sticherling C, Meinl C, May A, Schomig A (1997). Induction of cytokine expression in leukocytes by binding of thrombin-stimulated platelets. Circulation.

[CIT0155] Passacquale G, Vamadevan P, Pereira L, Hamid C, Corrigall V, Ferro A (2011). Monocyte-platelet interaction induces a pro-inflammatory phenotype in circulating monocytes. PLoS One.

[CIT0156] Rainger GE, Rowley AF, Nash GB (1998). Adhesion-dependent release of elastase from human neutrophils in a novel, flow-based model: Specificity of different chemotactic agents. Blood.

[CIT0157] Scheuerer B, Ernst M, Durrbaum-Landmann I, Fleischer J, Grage-Griebenow E, Brandt E, Flad HD, Petersen F (2000). The cxc-chemokine platelet factor 4 promotes monocyte survival and induces monocyte differentiation into macrophages. Blood.

[CIT0158] Nassar T, Sachais BS, Akkawi S, Kowalska MA, Bdeir K, Leitersdorf E, Hiss E, Ziporen L, Aviram M, Cines D (2003). Platelet factor 4 enhances the binding of oxidized low-density lipoprotein to vascular wall cells. J Biol Chem.

[CIT0159] Gleissner CA, Shaked I, Little KM, Ley K (2010). Cxc chemokine ligand 4 induces a unique transcriptome in monocyte-derived macrophages. J Immunol.

[CIT0160] Gleissner CA, Shaked I, Erbel C, Bockler D, Katus HA, Ley K (2010). Cxcl4 downregulates the atheroprotective hemoglobin receptor cd163 in human macrophages. Circ Res.

[CIT0161] Suzuki J, Hamada E, Shodai T, Kamoshida G, Kudo S, Itoh S, Koike J, Nagata K, Irimura T, Tsuji T (2013). Cytokine secretion from human monocytes potentiated by p-selectin-mediated cell adhesion. Int Arch Allergy Immunol.

[CIT0162] Seta N, Okazaki Y, Miyazaki H, Kato T, Kuwana M (2013). Platelet-derived stromal cell-derived factor-1 is required for the transformation of circulating monocytes into multipotential cells. PLoS One.

[CIT0163] Seta N, Kuwana M (2010). Derivation of multipotent progenitors from human circulating CD14+ monocytes. Exp Hematol.

[CIT0164] Spectre G, Zhu L, Ersoy M, Hjemdahl P, Savion N, Varon D, Li N (2012). Platelets selectively enhance lymphocyte adhesion on subendothelial matrix under arterial flow conditions. Thromb Haemost.

[CIT0165] von Hundelshausen P, Weber C (2007). Platelets as immune cells: Bridging inflammation and cardiovascular disease. Circ Res.

[CIT0166] Baigent C, Blackwell L, Collins R, Emberson J, Godwin J, Peto R, Buring J, Hennekens C, Kearney P, Meade T (2009). Aspirin in the primary and secondary prevention of vascular disease: Collaborative meta-analysis of individual participant data from randomised trials. Lancet.

[CIT0167] Eikelboom JW, Hirsh J, Spencer FA, Baglin TP, Weitz JI (2012). Antiplatelet drugs: Antithrombotic therapy and prevention of thrombosis. American college of chest physicians evidence-based clinical practice guidelines (9th ed). Chest.

[CIT0168] Patrono C, Andreotti F, Arnesen H, Badimon L, Baigent C, Collet JP, De Caterina R, Gulba D, Huber K, Husted S (2011). Antiplatelet agents for the treatment and prevention of atherothrombosis. Eur Heart J.

[CIT0169] Awtry EH, Loscalzo J (2000). Aspirin. Circulation.

[CIT0170] Li N, Hu H, Hjemdahl P (2003). Aspirin treatment does not attenuate platelet or leukocyte activation as monitored by whole blood flow cytometry. Thromb Res.

[CIT0171] Wallentin L (2009). P2y(12) inhibitors: Differences in properties and mechanisms of action and potential consequences for clinical use. Eur Heart J.

[CIT0172] Azar RR, Kassab R, Zoghbi A, Aboujaoude S, El-Osta H, Ghorra P, Germanos M, Salame E (2006). Effects of clopidogrel on soluble cd40 ligand and on high-sensitivity c-reactive protein in patients with stable coronary artery disease. Am Heart J.

[CIT0173] Heitzer T, Rudolph V, Schwedhelm E, Karstens M, Sydow K, Ortak M, Tschentscher P, Meinertz T, Boger R, Baldus S (2006). Clopidogrel improves systemic endothelial nitric oxide bioavailability in patients with coronary artery disease: Evidence for antioxidant and antiinflammatory effects. Arterioscler Thromb Vasc Biol.

[CIT0174] Klinkhardt U, Bauersachs R, Adams J, Graff J, Lindhoff-Last E, Harder S (2003). Clopidogrel but not aspirin reduces p-selectin expression and formation of platelet-leukocyte aggregates in patients with atherosclerotic vascular disease. Clin Pharmacol Ther.

[CIT0175] Quinn MJ, Bhatt DL, Zidar F, Vivekananthan D, Chew DP, Ellis SG, Plow E, Topol EJ (2004). Effect of clopidogrel pretreatment on inflammatory marker expression in patients undergoing percutaneous coronary intervention. Am J Cardiol.

[CIT0176] Vavuranakis M, Latsios G, Aggelis D, Bosinakou I, Karambelas I, Tousoulis D, Toutouzas K, Stefanadis C (2006). Randomized comparison of the effects of asa plus clopidogrel versus asa alone on early platelet activation in acute coronary syndromes with elevated high-sensitivity c-reactive protein and soluble CD40 ligand levels. Clin Ther.

[CIT0177] Xiao Z, Theroux P (2004). Clopidogrel inhibits platelet-leukocyte interactions and thrombin receptor agonist peptide-induced platelet activation in patients with an acute coronary syndrome. J Am Coll Cardiol.

[CIT0178] Antonino MJ, Mahla E, Bliden KP, Tantry US, Gurbel PA (2009). Effect of long-term clopidogrel treatment on platelet function and inflammation in patients undergoing coronary arterial stenting. Am J Cardiol.

[CIT0179] Braun OO, Johnell M, Varenhorst C, James S, Brandt JT, Jakubowski JA, Winters KJ, Wallentin L, Erlinge D, Siegbahn A (2008). Greater reduction of platelet activation markers and platelet-monocyte aggregates by prasugrel compared to clopidogrel in stable coronary artery disease. Thromb Haemost.

[CIT0180] Frelinger AL, Jakubowski JA, Li Y, Barnard MR, Fox ML, Linden MD, Sugidachi A, Winters KJ, Furman MI, Michelson AD (2007). The active metabolite of prasugrel inhibits ADP-stimulated thrombo-inflammatory markers of platelet activation: Influence of other blood cells, calcium, and aspirin. Thromb Haemost.

[CIT0181] Serebruany VL, Midei MG, Meilman H, Malinin AI, Lowry DR (2006). Platelet inhibition with prasugrel (cs-747) compared with clopidogrel in patients undergoing coronary stenting: The subset from the jumbo study. Postgrad Med J.

[CIT0182] Wallentin L, Becker RC, Budaj A, Cannon CP, Emanuelsson H, Held C, Horrow J, Husted S, James S, Katus H (2009). Ticagrelor versus clopidogrel in patients with acute coronary syndromes. N Engl J Med.

[CIT0183] Husted S, Storey RF, Harrington RA, Emanuelsson H, Cannon CP (2010). Changes in inflammatory biomarkers in patients treated with ticagrelor or clopidogrel. Clin Cardiol.

[CIT0184] Gawaz M, Langer H, May AE (2005). Platelets in inflammation and atherogenesis. J Clin Invest.

[CIT0185] Blann AD, Nadar SK, Lip GY (2003). The adhesion molecule p-selectin and cardiovascular disease. Eur Heart J.

[CIT0186] Bienvenu JG, Tanguay JF, Theoret JF, Kumar A, Schaub RG, Merhi Y (2001). Recombinant soluble p-selectin glycoprotein ligand-1-ig reduces restenosis through inhibition of platelet-neutrophil adhesion after double angioplasty in swine. Circulation.

[CIT0187] Hansen A, Kumar A, Wolf D, Frankenbergerova K, Filusch A, Gross ML, Mueller S, Katus H, Kuecherer H (2004). Evaluation of cardioprotective effects of recombinant soluble p-selectin glycoprotein ligand-immunoglobulin in myocardial ischemia-reperfusion injury by real-time myocardial contrast echocardiography. J Am Coll Cardiol.

[CIT0188] Hayward R, Campbell B, Shin YK, Scalia R, Lefer AM (1999). Recombinant soluble p-selectin glycoprotein ligand-1 protects against myocardial ischemic reperfusion injury in cats. Cardiovasc Res.

[CIT0189] Kumar A, Villani MP, Patel UK, Keith JC, Schaub RG (1999). Recombinant soluble form of psgl-1 accelerates thrombolysis and prevents reocclusion in a porcine model. Circulation.

[CIT0190] Wang K, Zhou X, Zhou Z, Tarakji K, Qin JX, Sitges M, Shiota T, Forudi F, Schaub RG, Kumar A (2002). Recombinant soluble p-selectin glycoprotein ligand-ig (rpsgl-ig) attenuates infarct size and myeloperoxidase activity in a canine model of ischemia-reperfusion. Thromb Haemost.

[CIT0191] Mertens P, Maes A, Nuyts J, Belmans A, Desmet W, Esplugas E, Charlier F, Figueras J, Sambuceti G, Schwaiger M (2006). Recombinant p-selectin glycoprotein ligand-immunoglobulin, a p-selectin antagonist, as an adjunct to thrombolysis in acute myocardial infarction. The p-selectin antagonist limiting myonecrosis (psalm) trial. Am Heart J.

[CIT0192] Tanguay JF, Krucoff MW, Gibbons RJ, Chavez E, Liprandi AS, Molina-Viamonte V, Aylward PE, Lopez-Sendon JL, Holloway DS, Shields K (2003). Efficacy of a novel p-selectin antagonist, rpsgl-ig for reperfusion therapy in acute myocardial infarction: The rapsody trial. J Am Coll Cardiol.

[CIT0193] Chelliah R, Lucking AJ, Tattersall L, Daga S, Beresford-Cleary NJ, Cortas K, Fox KA, Feuerstein GZ, Connolly TM, Newby DE (2009). P-selectin antagonism reduces thrombus formation in humans. J Thromb Haemost.

[CIT0194] Pamukcu B, Lip GY, Snezhitskiy V, Shantsila E (2011). The CD40-CD40L system in cardiovascular disease. Ann Med.

[CIT0195] Lutgens E, Cleutjens KB, Heeneman S, Koteliansky VE, Burkly LC, Daemen MJ (2000). Both early and delayed anti-CD40L antibody treatment induces a stable plaque phenotype. Proc Natl Acad Sci USA.

[CIT0196] Lutgens E, Gorelik L, Daemen MJ, de Muinck ED, Grewal IS, Koteliansky VE, Flavell RA (1999). Requirement for cd154 in the progression of atherosclerosis. Nat Med.

[CIT0197] Mach F, Schonbeck U, Sukhova GK, Atkinson E, Libby P (1998). Reduction of atherosclerosis in mice by inhibition of CD40 signalling. Nature.

[CIT0198] Schonbeck U, Sukhova GK, Shimizu K, Mach F, Libby P (2000). Inhibition of CD40 signaling limits evolution of established atherosclerosis in mice. Proc Natl Acad Sci USA.

[CIT0199] Couzin J (2005). Drug discovery. Magnificent obsession. Science.

[CIT0200] Chauhan AK, Kisucka J, Brill A, Walsh MT, Scheiflinger F, Wagner DD (2008). Adamts13: A new link between thrombosis and inflammation. J Exp Med.

[CIT0201] Pendu R, Terraube V, Christophe OD, Gahmberg CG, de Groot PG, Lenting PJ, Denis CV (2006). P-selectin glycoprotein ligand 1 and beta2-integrins cooperate in the adhesion of leukocytes to von willebrand factor. Blood.

[CIT0202] Firbas C, Siller-Matula JM, Jilma B (2010). Targeting von willebrand factor and platelet glycoprotein ib receptor. Expert Rev Cardiovasc Ther.

[CIT0203] Massberg S, Gawaz M, Gruner S, Schulte V, Konrad I, Zohlnhofer D, Heinzmann U, Nieswandt B (2003). A crucial role of glycoprotein VI for platelet recruitment to the injured arterial wall *in vivo*. J Exp Med.

[CIT0204] Bultmann A, Li Z, Wagner S, Peluso M, Schonberger T, Weis C, Konrad I, Stellos K, Massberg S, Nieswandt B (2010). Impact of glycoprotein vi and platelet adhesion on atherosclerosis – A possible role of fibronectin. J Mol Cell Cardiol.

[CIT0205] Ungerer M, Li Z, Baumgartner C, Goebel S, Vogelmann J, Holthoff HP, Gawaz M, Munch G (2013). The GPVI – FC fusion protein revacept reduces thrombus formation and improves vascular dysfunction in atherosclerosis without any impact on bleeding times. PLoS One.

[CIT0206] Goebel S, Li Z, Vogelmann J, Holthoff HP, Degen H, Hermann DM, Gawaz M, Ungerer M, Munch G (2013). The GPVI-FC fusion protein revacept improves cerebral infarct volume and functional outcome in stroke. PLoS One.

[CIT0207] Ungerer M, Rosport K, Bultmann A, Piechatzek R, Uhland K, Schlieper P, Gawaz M, Munch G (2011). Novel antiplatelet drug revacept (dimeric glycoprotein VI-FC) specifically and efficiently inhibited collagen-induced platelet aggregation without affecting general hemostasis in humans. Circulation.

[CIT0208] Owens AP, Mackman N (2011). Microparticles in hemostasis and thrombosis. Circ Res.

[CIT0209] Suzuki-Inoue K, Fuller GL, Garcia A, Eble JA, Pohlmann S, Inoue O, Gartner TK, Hughan SC, Pearce AC, Laing GD (2006). A novel syk-dependent mechanism of platelet activation by the c-type lectin receptor CLEC-2. Blood.

[CIT0210] Suzuki-Inoue K, Kato Y, Inoue O, Kaneko MK, Mishima K, Yatomi Y, Yamazaki Y, Narimatsu H, Ozaki Y (2007). Involvement of the snake toxin receptor CLEC-2, in podoplanin-mediated platelet activation, by cancer cells. J Biol Chem.

[CIT0211] Herzog BH, Fu J, Wilson SJ, Hess PR, Sen A, McDaniel JM, Pan Y, Sheng M, Yago T, Silasi-Mansat R (2013). Podoplanin maintains high endothelial venule integrity by interacting with platelet CLEC-2. Nature.

